# Efficacy of commercial vaccines against newly emerging avian influenza H5N8 virus in Egypt

**DOI:** 10.1038/s41598-018-28057-x

**Published:** 2018-06-26

**Authors:** Ahmed Kandeil, Jamal S. M. Sabir, Ahmed Abdelaal, Ehab H. Mattar, Ahmed N. El-Taweel, Mumdooh J. Sabir, Ahmed Aly Khalil, Richard Webby, Ghazi Kayali, Mohamed A. Ali

**Affiliations:** 1 Center of Scientific Excellence for Influenza Viruses, National Research Center, Giza, Egypt; 2 Biotechnology Research Group, Department of Biological Sciences, Faculty of Science, King Abdulaziz University (KAU), Jeddah, 21589 Saudi Arabia; 3 Faculty of Biotechnology, October University of Modern Sciences and Arts (MSA), Cairo, Egypt; 4 Department of Information Technology, Faculty of Computing and Information Technology, King Abdulaziz University, Jeddah, Saudi Arabia; 5 Veterinary Serum and Vaccine Research Institute, Cairo, Egypt; 6 Department of Infectious Diseases, St. Jude Children’s Research Hospital, Memphis, TN United States; 7 Department of Epidemiology, Human Genetics, and Environmental Sciences, University of Texas Health Sciences Center, Houston, Texas USA; 8 Human Link, Hazmieh, Lebanon

## Abstract

The newly emerging, highly pathogenic avian influenza (HPAI) H5N8 virus of clade 2.3.4.4 was recently detected in wild birds and domestic poultry in Egypt in the 2016/2017 winter season. Vaccination based on commercial H5 vaccines is used as an essential control strategy in Egyptian poultry. Here, we studied the efficacy of the eight most common commercial H5 poultry vaccines in the Egyptian market and compared them with an experimental vaccine based on the Egyptian LPAI H5N8 virus that was prepared by using reverse genetics. The experimental vaccine and Re-5 commercial vaccine were able to completely protect chickens and significantly reduce virus shedding. Our results indicate that most of the commercial poultry H5 vaccines used in the present study were ineffective because the seed viruses in these vaccines are genetically distinct from the H5N8 viruses currently circulating in Egypt. Although some of the commercial vaccines protected chickens from mortality, they failed to prevent chickens from shedding the virus. Accordingly, we recommend updating and reinforcing the H5N8 prevention and control strategies in Egypt. The vaccination strategy should be reconsidered based on currently circulating viruses.

## Introduction

Since 2006, clade 2.2.1 of highly pathogenic avian influenza (HPAI) H5N1 viruses has been widely circulating in Egypt, causing massive economic losses in the Egyptian poultry industry^1^. The Egyptian veterinary authorities tried to apply a comprehensive response plan to control the spread of the virus but had limited success due to the presence of gaps in implementation. The control plan included establishing biosafety and biosecurity measures in poultry farms, increasing public awareness through the media, culling infected poultry, restricting poultry movement between governorates, and implementing emergency vaccination^2,3^. Despite these efforts, the H5N1 virus became enzootic, and the virus evolved into 3 antigenically distinct subclades (2.2.1.1, 2.2.1.1a, and 2.2.1.2)^4^.

At least 24 commercial avian influenza H5 vaccines have been licensed for use for H5N1 prophylaxis in Egyptian poultry^3^. The vaccine seed strains of these commercial products were based either on classic low pathogenicity H5NX viruses or on H5N1 reassortants harboring two surface glycoprotein (HA and NA) genes of H5N1 (clade 0, 1, 2.2.1, 2.3.2, and 2.3.4) viruses in the genetic background of the A/Puerto Rico/8/1934 (H1N1) strain^3^. Interestingly, some of the tested vaccines were immunogenic and protected chickens challenged with the Egyptian highly pathogenic H5N1 viruses under laboratory conditions^5–7^. However, the H5N1 and H5N2 experimentally prepared vaccines fared poorly when tested in the field in Egypt^8^. Previous studies on the cross-reactivity of commercial H5 poultry vaccines against H5N1 Egyptian isolates in the field in Egypt showed that only one vaccine based on an Egyptian H5N1 virus induced high cross-reactive antibody titers^9^.

The emerging clade 2.3.4.4 HPAI H5N8 virus was first detected in a live bird market in China in 2010^10^. By 2014, H5N8 viruses had caused different outbreaks in domestic poultry and wild birds in South Korea. Several outbreaks were subsequently recorded either in wild or domesticated birds in many Eurasian and North American countries between 2014 and 2017. The HPAI H5N8 virus has been recently detected in wild birds and domestic poultry in Egypt^11,12^.

However, little is known about the efficacy and cross reactivity of commercial vaccines against the HPAI H5N8 virus. Herein, we assessed the efficacy of eight commercial vaccines based on different lineages of AI/H5 viruses against the newly emerging HPAI H5N8 virus in Egypt.

## Results

Eight of the most common commercial AIV vaccines and an experimental inactivated vaccine based on the Egyptian LPAI H5N8 virus were used to vaccinate 9 chicken groups. All the collected swabs from the chickens up to 6 weeks of age were tested negative by RT-PCR, suggesting that the chickens were not exposed to natural AIV infection during the immune response follow up period. The maternal antibodies at one week of age cross-reacted with H5N1, H5N8, and H9N2 viruses, with log_2_ mean titers of 4.5, 1.7, and 3, respectively. At 2 weeks of age (day of vaccination), the reactivities against H5N1, H5N8, and H9N2 viruses were 4, 3, and 3 log_2_ mean titer, respectively. Serological responses to different types of vaccines were assessed weekly until 4 weeks post vaccination (wpv).

**Figure 1:**
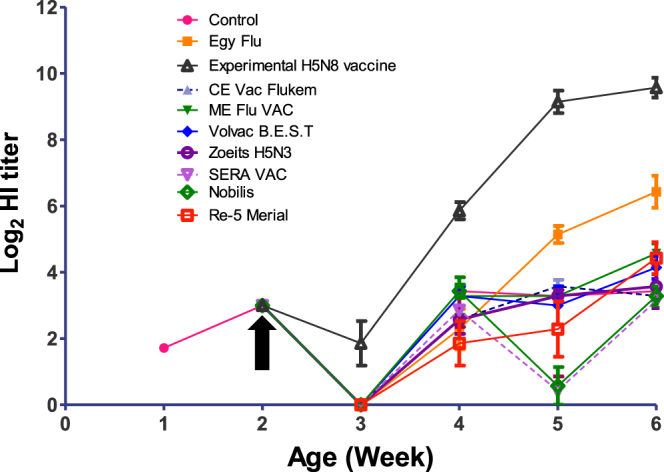
Profile of weekly log_2_ HI antibody responses of chickens vaccinated with eight commercial H5 vaccines and the experimental RG H5N8 vaccine against the Egyptian H5N8 virus. Chickens were vaccinated at 2 weeks of age (indicated by an arrow). Data are presented as the mean ± standard deviation.

Chickens vaccinated with the experimental vaccine showed significantly higher antibody titers against the homologous virus at two wpv with a mean HI titer of 5.8 log_2_ significantly higher than the titer of the control group (p < 0.01, Fig. 1). The mean HI titer of chickens vaccinated with the experimental vaccine increased notably to 9.1 log_2_ at 3 wpv and continued increasing to reach 9.5 log_2_ at 4 wpv (p < 0.001 compared to the control group).

No tested commercial vaccines induced any significant HI titers (p > 0.05) against the heterologous H5N8 virus until 3 wpv. At 4 wpv, only Egyflu had a significant cross-reactive log_2_ HI titer of 6.4 (p ≤ 0.001), whereas the other vaccines had no significant HI titer (p > 0.05, Fig. 1).

The HPAI A/duck/Egypt/F13666A/2017(H5N8) virus isolated from domestic poultry was lethal to unvaccinated chickens. It showed 100% mortality at four days post infection, with clinical signs of AIV infection including cyanotic combs and wattle, hemorrhagic legs, lethargy, anorexia, and diarrhea.

**Figure 2:**
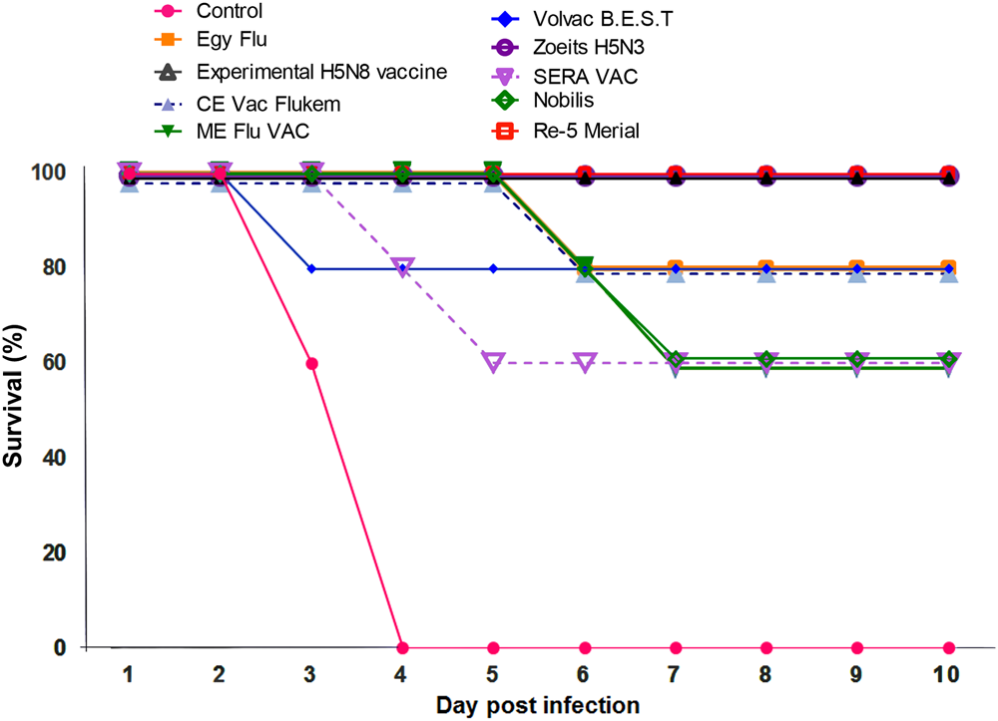
Survival curves of unimmunized and immunized poultry with tested vaccines after challenge with 0.5 mL of 10^7.5^ EID_50_ of the HP H5N8 virus.

All chickens vaccinated with the Re-5 Merial, Zoetis H5N3, and experimental H5N8 vaccines survived until 10 days post infection with no clinical signs of AIV infection (Fig. 2). In contrast, chickens vaccinated with the remaining commercial vaccines succumbed to the viral infection, with mortality rates ranging from 60% for the Nobilis, ME Flu VAC and SER-VAC vaccines to 80% for the CEVac Flukem, Egyflu and Volvac (B.E.S.T.) vaccines, with milder clinical signs of AIV infection than those in unvaccinated chickens. Nevertheless, these groups showed significantly higher survival rates compared with the survival rate of the unvaccinated chicken group (p < 0.01).

**Figure 3:**
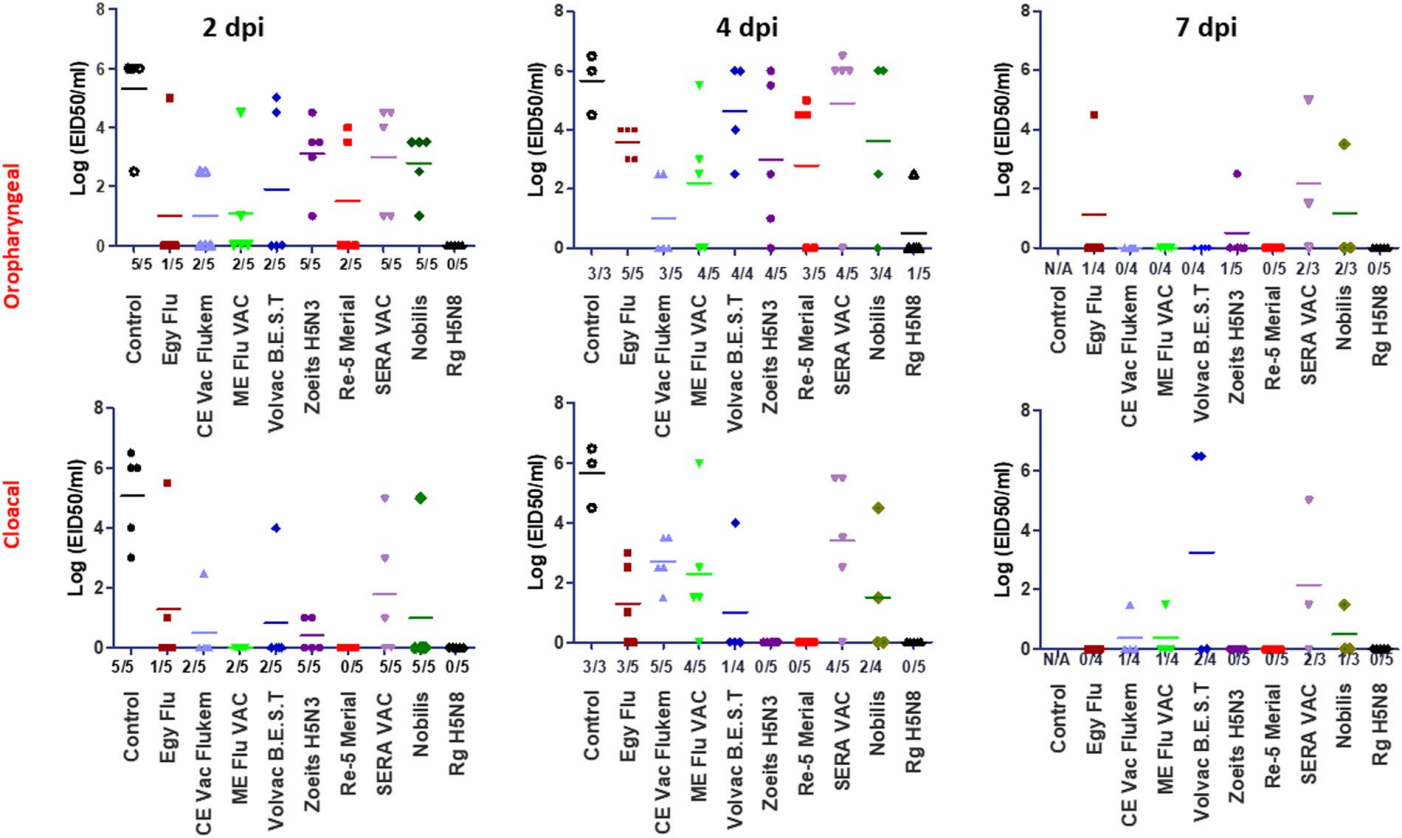
Titration of viral shedding from collected oropharyngeal and cloacal swabs after challenge of unimmunized and immunized chickens with the Egyptian HPAIV H5N8 isolate at 2, 4 and 7 dpi. Five chickens from each group were individually infected with the HP H5N8 virus. Virus shedding was monitored by titration of log_10_ EID_50_/mL for each collected sample from live animals. Each dot represents the viral titer of each chicken. The bar for each vaccine is the mean of viral shedding in the group. The detection limit was <1 log_10_ EID_50_/0.1 mL.

Virus shedding was monitored by the quantification of viral titers in oropharyngeal and cloacal swabs collected at 2, 4, and 7 days post infection. The virus titers in the oropharyngeal swabs were higher than those in the cloacal swabs. Virus shedding peaked at the fourth day post infection. In the unvaccinated chickens, the virus was detected in both oropharyngeal and cloacal swabs at day 2, with mean titers of 5.3 and 5 log_10_ EID_50_/mL, respectively. The virus was isolated from the three surviving chickens of the control group at day 4 post infection with the same virus titer of 5.66 log_10_ EID_50_/mL in the oral and cloacal swabs. All commercial vaccines reduced viral shedding in challenged vaccinated chickens. No virus was recovered from the oral and cloacal swabs collected from chickens vaccinated with the experimental vaccine at days 2 and 7 post infection. The virus was recovered from only one oral swab at day 4 post infection with a titer of 2.5 log_10_ EID_50_/mL (Fig. 3).

No virus was detected in the cloacal swabs of chickens vaccinated with Re-5 Merial. However, it was detected in the oropharyngeal swabs at days 2 and 4 with titers of 1.5 and 2.8 log_10_ EID_50_/mL, respectively. Another commercial vaccine, Zoetis H5N3, provided protection against morbidity/mortality to vaccinated chickens, but virus shedding was observed in oropharyngeal swabs at days 2, 4, and 7 post infection with virus titers of 3.1, 3, and 0.5 log_10_EID_50_/mL, respectively.

Oropharyngeal shedding at 4 dpi was detected in chickens vaccinated with Egyflu (5/5 chickens), CEvac Flukem (3/5 chickens), ME Flu VAC (4/5 chickens), Volvac (B.E.S.T.) (4/4 chickens), Nobilis (3/4 chickens), and SERA-VAC (4/5 chickens) with virus titers of 3.6, 1, 2.2, 4.6, 4.9, and 3.6 log_10_ EID_50_/mL, respectively. The virus was detected in the cloacal swabs of vaccinated chickens at 4 dpi with Egyflu (3/5 chickens), CEVac Flukem (3/5 chickens), ME Flu VAC (4/5 chickens), Volvac (B.E.S.T.) (4/4 chickens), Nobilis (3/4 chickens), and SERA-VAC (4/5 chickens) with titers of 1.3, 2.7, 2.3, 1, 3.6 and 4.9 log_10_ EID_50_/mL, respectively.

## Discussion

Multiple factors influence poultry vaccine efficacy. One of these critical factors is the genetic and antigenic matching between the circulating viruses and commercial vaccine strains^13^. According to the manual for vaccine evaluation by the World Organization for Animal Health (OIE), an effective poultry vaccine should protect at least 80% of vaccinated chickens from death and should reduce viral shedding after a challenge infection.

Inactivated AI/H5 vaccines based on phylogenetically distinct seed strains have been used in Egypt since 2006. Several experimental studies were conducted in chickens to evaluate the efficacy of different types of commercial vaccines against challenges with different Egyptian H5N1 viruses of clades 2.2.1, 2.2.1.1, 2.2.1.1a, and 2.2.1.2. The serological results showed that different types of commercial AI vaccines provided variable reactivity against the previously described antigens of Egyptian H5N1 viruses (isolates from 2006 to 2009, clade 2.2.1), but that reactivity declined with recent circulating viruses of clade 2.2.1.2^3^. Except for Egyptian H5N1-based vaccines, the different types of commercial vaccines used in Egypt did not confer full protection against different clades of Egyptian H5N1 viruses^7,9,14,15^.

The HPAI H5N8 virus was recently detected in wild birds and domestic poultry in Egypt^11,12^. The most dominant control strategy for H5N1 in Egypt is vaccination using commercial AI/H5 vaccines. However, the detection of clade 2.3.4.4 H5N8 viruses in poultry in 2017 revealed the need to readdress the ability of the commercial H5 vaccine used in Egypt to protect poultry against the newly emerging H5N8 virus. We tested the efficacy of the eight most common commercial vaccines against a challenge with the HPAI A/duck/Egypt/F13666A/2017 (H5N8) virus (clade 2.3.4.4) in white leghorn chickens. Maternal antibodies were previously investigated as one of the possible factors affecting vaccine failure in chickens^6,9,16^. To exclude the interference of maternal antibodies with the different tested vaccines, chickens were vaccinated at 2 weeks of age. The experimental homologous H5N8 vaccine provided the best protection against a challenge with the clade 2.3.4.4 virus. The sera of chickens vaccinated with the Re-5 Merial, Zoetis, EgyFlu, CEVac Flukem and Volvac (B.E.S.T.) vaccines showed reduced cross-reactivity against the Egyptian H5N8 virus and provided ≥80% protection, while the Nobilis, ME Flu VAC, and SERA-VAC vaccines did not reach the protection limit recommended by the OIE. The Re-5 Merial (based on a clade 2.3.4 H5N1 virus) vaccine protected the chickens from mortality and reduced virus shedding. Most of the commercial vaccines protected chickens from mortality but did not reduce or prevent virus shedding. This suggests that the circulating H5N8 viruses may evade vaccine protection. The genetic dissimilarity and poor reactivity between the H5 commercial vaccines used in Egypt and the currently circulating H5N8 viruses proves that the vaccines might not be effective in the field or may introduce only partial protection and thus could lead to vaccine-induced escape mutant strains.

## Materials and Methods

### Vaccines and Viruses

Eight of the most common commercially available vaccines during 2017 were purchased from poultry vaccine distributors in Egypt (Table 1). The vaccines were based on several strains licensed for the control of the HPAI H5N1 virus in poultry. The Volvac® B.E.S.T. (Baculovirus Expression System Technology) is the commercial bivalent vaccine for AI/H5 and Newcastle disease viruses and is based on a recombinant baculovirus propagated in insect cells. The HA of the Volvac® B.E.S.T. vaccine originated from the A/duck/China/E319-2/03 (H5N1, clade 2.3.2) virus. Seven commercial oil-adjuvant inactivated vaccines were based on either reverse genetically engineered reassortant (RG) AI/H5 viruses (clades 1, 2.2.1.1, 2.2.1.2, and 2.3.4) or classic H5N2 vaccine seed strains.

**Table 1:** List of H5 commercial vaccines used for the immunization of poultry against H5N8.

Vaccine trade name	Virus used	Lineage	Manufacturer, Country	HA nucleotide sequence % similarity to Egyptian H5N8
Nobilis Influenza H5N2	A/duck/Potsdam/1402-6/1986(H5N2)	Classic	Intervet, The Netherlands	87
CEVac Flukem	A/chicken/Mexico/232/1994 (H5N2)	Classic	Ceva, Mexico	80
Zoetis H5N3	A/chicken/Vietnam/C58/2004 (H5N3)	Clade 1	Zoetis, USA	91
EgyFlu	RG A/chicken/Egypt/18-H/2009 (H5N1)	Clade 2.2.1.1	Harbin Veterinary Research Institute, China	89
ME Flu VAC	RG A/duck/ Egypt /M2583D /2010 (H5N1)	Clade 2.2.1.2	ME-VAC, Egypt	90
SERA-VAC	RG A/chicken/Egypt/M2583D/2010(H5N1)	Clade 2.2.1.2	Veterinary Serum and Vaccine Institute, Egypt	90
Volvac (B.E.S.T.)	A/duck/China/E319-2/2003 (H5N1) + ND	Clade 2.3.2	Boehringer Ingelheim, Mexico	93
Reassortant AIV (strain Re-5) Re-5, Merial	RG A/duck/Anhui/1/2006(H5N1) (Re-5)	Clade 2.3.4	Merial, China	94

The HPAI H5N8 virus, A/green winged teal/Egypt/871/2016 (clade 2.3.4.4), was used to prepare an experimental vaccine. A plasmid-based reverse genetics system was applied using six internal genes of the A/Puerto Rico/8/34(H1N1) virus, the LP form of HA (accession no. MF037851.1) and the NA (accession no. MF037848.1) genes of the H5N8 virus^17^. The generated RG vaccine strain was propagated in 10-day-old specific pathogen-free embryonated chicken eggs (SPF-ECE) (Koum Oshiem SPF Chicken Farm, Fayoum, Egypt) for 3 passages of 48 h each and did not result in embryo death. To create the experimental vaccine, allantoic fluid containing the RG H5N8 virus (titer = 7 log_2_ HA/50 µL) was inactivated by the addition of 0.1% formalin and mixed with Montanide ISA 70 VG (Seppic, France) in the ratio recommended by the manufacturer (30 antigen/70 adjuvant). The LPAI A/chicken/Egypt/S10489C/2015(H9N2) and A/chicken/Egypt/D10552B/2015 (H5N1) viruses were used as antigens to detect antibodies against H9N2 and H5N1 viruses, respectively^18^. The plaque-purified HPAI A/duck/Egypt/F13666A/2017(H5N8) virus was used for laboratory challenge experiments.

### Immunization of chickens and serological assays

A total of 100 one-week-old Lohmann White chicks were divided into 10 groups (10 chicks/group). Serum samples collected from 10 randomly selected chicks were tested for H5N1, H5N8, and H9N2 antibodies resulting from maternally transmitted immunity at ages one and two weeks by hemagglutination inhibition (HI) assay using chicken RBCs^18^. Nine groups of chickens received 0.5 mL/chick of a vaccine by intramuscular injection into the thigh at 14 days old. One group was used as an unvaccinated control. Once a week for the first three weeks post vaccination (wpv), blood samples were withdrawn from 7 chickens in each group to evaluate antibody titers against the H5N8 virus by HI. Chickens were monitored daily for morbidity and mortality. Cloacal and oral swabs were obtained weekly from each group post vaccination to monitor influenza A virus infection.

### Challenge infection and determination of virus shedding

From each group, five animals were randomly selected at four wpv. Each of the five was infected with a 0.5 mL total dose containing 10^7.5^ EID_50_ HP H5N8 challenge virus via intranasal and intratracheal routes (0.25 mL/route). Birds were then monitored daily for morbidity and mortality until 10 days post infection. Cloacal and oral swabs were obtained from each bird at 2, 4, and 7 days post infection for virus shedding titration in each bird in different groups by calculating the EID_50_ per 1 mL of virus. All animal experiments were approved by the Ethics Committee of the National Research Center, Egypt. Experimental infection was performed at biosafety level 3 negative-pressure chicken isolators (PLAS LABS) and under controlled laboratory and biosafety conditions. Any chickens that showed a rapid onset of paralysis, disorientation, reluctance to feed, lethargy and loss of body weight was culled as the humane endpoint.

### Statistical analyses

Statistical analyses were performed with GraphPad Prism V5 (GraphPad Inc., CA, USA). An ANOVA with Tukey’s post hoc test was used to compare antibody titers and viral shedding yields. Differences were considered statistically significant at p ≤ 0.05.

### Ethical approval

All animal experiments were conducted in strict accordance with and adherence to the relevant policies regarding animal handling as mandated under international, national, and/or institutional guidelines for the care of animals and were approved by the Research Ethical Committee at the National Research Center, Cairo, Egypt. This article does not contain any studies with human participants performed by any of the authors.
